# Supplementary material for: Genomic Relatedness Strengthens Genetic Connectedness Across Management Units
Source: G3 (Bethesda). 2017 Aug 31;7(10):3543–56. doi: 10.1534/g3.117.300151 (PMC5633401; doi:10.1534/g3.117.300151)
Supplement: Supplementary file 1 [file 3543FileS1.pdf]

Text S1

We further investigated how the $\mathbf{G}$ or $\mathbf{G_s}$ increased connectedness across management units using PEVD and r by examining the specific components in the scaled PEV matrix. For instance, disconnected "19F" and "13C" management units contained five full-sib individuals each. The following matrix contains the pedigree-based PEV for the 10 individuals.

$$\begin{bmatrix} 0.73 & 0.57 & 0.57 & 0.57 & 0.57 & 0.00 & 0.00 & 0.00 & 0.00 & 0.00 \\ 0.57 & 0.73 & 0.57 & 0.57 & 0.57 & 0.00 & 0.00 & 0.00 & 0.00 & 0.00 \\ 0.57 & 0.57 & 0.73 & 0.57 & 0.57 & 0.00 & 0.00 & 0.00 & 0.00 & 0.00 \\ 0.57 & 0.57 & 0.57 & 0.73 & 0.57 & 0.00 & 0.00 & 0.00 & 0.00 & 0.00 \\ 0.57 & 0.57 & 0.57 & 0.57 & 0.73 & 0.00 & 0.00 & 0.00 & 0.00 & 0.00 \\ 0.00 & 0.00 & 0.00 & 0.00 & 0.00 & 0.55 & 0.39 & 0.39 & 0.39 & 0.39 \\ 0.00 & 0.00 & 0.00 & 0.00 & 0.00 & 0.39 & 0.55 & 0.39 & 0.39 & 0.39 \\ 0.00 & 0.00 & 0.00 & 0.00 & 0.00 & 0.39 & 0.39 & 0.55 & 0.39 & 0.39 \\ 0.00 & 0.00 & 0.00 & 0.00 & 0.00 & 0.39 & 0.39 & 0.39 & 0.55 & 0.39 \\ 0.00 & 0.00 & 0.00 & 0.00 & 0.00 & 0.39 & 0.39 & 0.39 & 0.39 & 0.55 \end{bmatrix}$$

$$\underbrace{\qquad\qquad\qquad\qquad}_{19F}\quad\underbrace{\qquad\qquad\qquad\qquad}_{13C}$$

The first five individuals belong to "19F" and the remaining individuals belong to "13C". Because there are no full-sib pairs across management units, off-diagonals are all zero. Thus, PEVD and r of two individuals across management units are $0.73+0.55-(2\times 0) = 1.28$ and $0/(\sqrt{0.73}\times\sqrt{0.55}) = 0$, respectively.

When the **A** matrix is replaced with the **G** matrix, the PEV matrix becomes

$$
\begin{bmatrix}
0.30 & 0.19 & 0.19 & 0.19 & 0.19 & -0.01 & -0.01 & -0.01 & -0.01 & -0.01 \\
0.19 & 0.32 & 0.21 & 0.21 & 0.19 & -0.01 & -0.02 & -0.02 & -0.01 & -0.01 \\
0.19 & 0.21 & 0.32 & 0.19 & 0.20 & -0.01 & -0.01 & -0.01 & -0.01 & -0.01 \\
0.19 & 0.21 & 0.19 & 0.30 & 0.19 & -0.01 & -0.01 & -0.02 & -0.01 & -0.01 \\
0.19 & 0.19 & 0.20 & 0.19 & 0.30 & -0.01 & -0.01 & -0.02 & -0.01 & -0.01 \\
-0.01 & -0.01 & -0.01 & -0.01 & -0.01 & 0.24 & 0.14 & 0.14 & 0.15 & 0.14 \\
-0.01 & -0.02 & -0.01 & -0.01 & -0.01 & 0.14 & 0.24 & 0.16 & 0.14 & 0.13 \\
-0.01 & -0.02 & -0.01 & -0.02 & -0.02 & 0.14 & 0.16 & 0.26 & 0.14 & 0.14 \\
-0.01 & -0.01 & -0.01 & -0.01 & -0.01 & 0.15 & 0.14 & 0.14 & 0.25 & 0.14 \\
-0.01 & -0.01 & -0.01 & -0.01 & -0.01 & 0.14 & 0.13 & 0.14 & 0.14 & 0.24
\end{bmatrix}.
$$

$$\underbrace{\qquad\qquad\qquad\qquad}_{19F} \qquad \underbrace{\qquad\qquad\qquad\qquad}_{13C}$$

Average genomic relationships within management units were 0.419 and 0.440 for individuals in "19F" and "13C", respectively, whereas across management unit genomic relationships were -0.09. The off-diagonals of zeros in pedigree-based PEV were replaced with small negative values. Although the diagonal elements within management units are not all equal because of Mendelian sampling, PEVD between the first individuals from respective management units are $0.30 + 0.24 - (2 \times -0.01) = 0.56$. Given that off-diagonal elements are negligible, the rate of PEVD reduction from shifting from **A** to **G** is almost 50% with the most of difference coming from decreased PEV in the diagonals. Specifically, the rates of PEV reduction (diagonals) from **A** to **G** were 59% and 56% for the first individuals in "19F" and "13C", respectively. The rates of PEC reduction cannot be defined since all of the off-diagonal elements are zeros in **A**. Note that the r statistic using the **G** matrix does not yield increased estimates of connectedness compared with that using **A** because

$-0.01/(\sqrt{(}0.30) \times \sqrt{0.24}) = -0.04$. Now consider the $\mathbf{G_s}$ matrix

$$\underbrace{\begin{bmatrix} 0.68 & 0.56 & 0.56 & 0.55 & 0.56 & 0.32 & 0.32 & 0.32 & 0.32 & 0.32 \\ 0.56 & 0.69 & 0.58 & 0.57 & 0.56 & 0.32 & 0.31 & 0.31 & 0.32 & 0.32 \\ 0.56 & 0.58 & 0.69 & 0.56 & 0.56 & 0.32 & 0.32 & 0.32 & 0.32 & 0.32 \\ 0.55 & 0.57 & 0.56 & 0.67 & 0.56 & 0.32 & 0.32 & 0.31 & 0.32 & 0.32 \\ 0.56 & 0.56 & 0.56 & 0.56 & 0.67 & 0.32 & 0.32 & 0.31 & 0.32 & 0.32 \\ 0.32 & 0.32 & 0.32 & 0.32 & 0.32 & 0.61 & 0.50 & 0.50 & 0.51 & 0.49 \\ 0.32 & 0.31 & 0.32 & 0.32 & 0.32 & 0.50 & 0.61 & 0.51 & 0.49 & 0.48 \\ 0.32 & 0.31 & 0.32 & 0.31 & 0.31 & 0.50 & 0.51 & 0.63 & 0.50 & 0.50 \\ 0.32 & 0.32 & 0.32 & 0.32 & 0.32 & 0.51 & 0.49 & 0.50 & 0.62 & 0.49 \\ 0.32 & 0.32 & 0.32 & 0.32 & 0.32 & 0.49 & 0.48 & 0.50 & 0.49 & 0.60 \end{bmatrix}}_{19F \qquad\qquad 13C}.$$

Here the PEV matrix within management units more closely resembles those from pedigree-based PEV. In addition, the negative elements across management units PEV were replaced with positive values. With use of $\mathbf{G_s}$, PEVD and r between the first individuals from the two management units are $0.68 + 0.61 - (2 \times 0.32) = 0.65$ and $0.32/(\sqrt{0.68} \times \sqrt{0.61}) = 0.50$, respectively. When the scaled genomic relationship matrix $\mathbf{G_s}$ is used, r yields an increased connectedness estimate as compared to using pedigree-based relationships.

Likewise, a subsequent example with "19F" and "36F" which can be viewed as connected management units (Figure 1). Each management unit contains five full-sibs as in the previous case.

The following matrix of pedigree-based PEV includes these 10 individuals.

$$\begin{bmatrix} 0.73 & 0.57 & 0.57 & 0.57 & 0.57 & 0.50 & 0.50 & 0.50 & 0.50 & 0.50 \\ 0.57 & 0.73 & 0.57 & 0.57 & 0.57 & 0.50 & 0.50 & 0.50 & 0.50 & 0.50 \\ 0.57 & 0.57 & 0.73 & 0.57 & 0.57 & 0.50 & 0.50 & 0.50 & 0.50 & 0.50 \\ 0.57 & 0.57 & 0.57 & 0.73 & 0.57 & 0.50 & 0.50 & 0.50 & 0.50 & 0.50 \\ 0.57 & 0.57 & 0.57 & 0.57 & 0.73 & 0.50 & 0.50 & 0.50 & 0.50 & 0.50 \\ 0.50 & 0.50 & 0.50 & 0.50 & 0.50 & 0.73 & 0.57 & 0.57 & 0.57 & 0.57 \\ 0.50 & 0.50 & 0.50 & 0.50 & 0.50 & 0.57 & 0.73 & 0.57 & 0.57 & 0.57 \\ 0.50 & 0.50 & 0.50 & 0.50 & 0.50 & 0.57 & 0.57 & 0.73 & 0.57 & 0.57 \\ 0.50 & 0.50 & 0.50 & 0.50 & 0.50 & 0.57 & 0.57 & 0.57 & 0.73 & 0.57 \\ \underbrace{0.50 \quad 0.50 \quad 0.50 \quad 0.50 \quad 0.50}_{19F} & & & & & \underbrace{0.57 \quad 0.57 \quad 0.57 \quad 0.57 \quad 0.73}_{36F} & & & & \end{bmatrix}$$

The first five individuals belong to "19F" and the remaining individuals belong to "36F". In this case, off-diagonals are non-zero due to the presence of shared full-sib information across management units. Here PEVD and r of two individuals is $0.73 + 0.73 - (2 \times 0.50) = 0.46$ and $0.50/(\sqrt{0.73} \times \sqrt{0.73}) = 0.68$, respectively. Relative to the pedigree-based non-full-sib comparison, a significant increase in connectedness was observed. The majority of the increase in connectedness is due to increased PEC between individuals.

The following is the PEV matrix when pedigree is substituted with the genome-wide markers

from **G**.

$$\begin{bmatrix} 0.30 & 0.19 & 0.19 & 0.19 & 0.19 & 0.19 & 0.18 & 0.18 & 0.18 & 0.19 \\ 0.19 & 0.32 & 0.21 & 0.21 & 0.19 & 0.18 & 0.18 & 0.18 & 0.19 & 0.19 \\ 0.19 & 0.21 & 0.32 & 0.19 & 0.20 & 0.18 & 0.18 & 0.18 & 0.20 & 0.19 \\ 0.19 & 0.21 & 0.19 & 0.30 & 0.19 & 0.18 & 0.17 & 0.19 & 0.18 & 0.18 \\ 0.19 & 0.19 & 0.20 & 0.19 & 0.30 & 0.18 & 0.17 & 0.18 & 0.18 & 0.19 \\ 0.19 & 0.18 & 0.18 & 0.18 & 0.18 & 0.31 & 0.20 & 0.19 & 0.21 & 0.20 \\ 0.18 & 0.18 & 0.18 & 0.17 & 0.17 & 0.20 & 0.31 & 0.18 & 0.21 & 0.20 \\ 0.18 & 0.18 & 0.18 & 0.19 & 0.18 & 0.19 & 0.18 & 0.29 & 0.20 & 0.20 \\ 0.18 & 0.19 & 0.20 & 0.18 & 0.18 & 0.21 & 0.21 & 0.20 & 0.32 & 0.21 \\ \underbrace{0.19 \quad 0.19 \quad 0.19 \quad 0.18 \quad 0.19}_{19F} & & & & & \underbrace{0.20 \quad 0.20 \quad 0.20 \quad 0.21 \quad 0.31}_{36F} \end{bmatrix}.$$

Average genomic relationship within "36F" was 0.465 whereas average genomic relationship across "19F" was 0.419. The off-diagonals no longer have small negative values and all elements of PEV were reduced in comparison to those using **A**. Here PEVD and r between the first individuals from the two management units are $0.30 + 0.31 - (2 \times 0.19) = 0.23$ and $0.19/(\sqrt{0.30} \times \sqrt{0.31}) = 0.62$, respectively. Again, while genomic information increased estimates of connectedness as measured by PEVD, that was not the case for r. The reduction in PEVD from **A** to **G** was about 50% and both diagonals and off-diagonals contributed to increasing connectedness estimates. In particular, the rates of PEV reduction (diagonals) from **A** to **G** were 59% and 58% for the first individuals in "19F" and "36F", respectively. The reduction in PEC due to the use of **G** was 62% for these two individuals, which was larger than the reduction of the diagonals. This contributed to the unexpected results for r because this statistic is based on a ratio. Now consider use of the scaled

genomic relationship matrix, $\mathbf{G_s}$, which yielded the following PEV matrix

$$\underbrace{\begin{bmatrix} 0.68 & 0.56 & 0.56 & 0.55 & 0.56 \\ 0.56 & 0.69 & 0.58 & 0.57 & 0.56 \\ 0.56 & 0.58 & 0.69 & 0.56 & 0.56 \\ 0.55 & 0.57 & 0.56 & 0.67 & 0.56 \\ 0.56 & 0.56 & 0.56 & 0.56 & 0.67 \\ 0.55 & 0.54 & 0.54 & 0.54 & 0.55 \\ 0.55 & 0.54 & 0.55 & 0.53 & 0.53 \\ 0.54 & 0.54 & 0.54 & 0.55 & 0.54 \\ 0.54 & 0.55 & 0.56 & 0.54 & 0.54 \\ 0.56 & 0.55 & 0.55 & 0.55 & 0.55 \end{bmatrix}}_{19F}\underbrace{\begin{bmatrix} 0.55 & 0.55 & 0.54 & 0.54 & 0.56 \\ 0.54 & 0.54 & 0.54 & 0.55 & 0.55 \\ 0.54 & 0.55 & 0.54 & 0.56 & 0.55 \\ 0.54 & 0.53 & 0.55 & 0.54 & 0.55 \\ 0.55 & 0.53 & 0.54 & 0.54 & 0.55 \\ 0.69 & 0.57 & 0.55 & 0.58 & 0.56 \\ 0.57 & 0.68 & 0.55 & 0.57 & 0.56 \\ 0.55 & 0.55 & 0.67 & 0.57 & 0.57 \\ 0.58 & 0.57 & 0.57 & 0.70 & 0.58 \\ 0.56 & 0.56 & 0.57 & 0.58 & 0.69 \end{bmatrix}}_{36F}.$$

The PEV matrix including both within and across management units are more analogous to those of the pedigree-based PEV. Here PEVD and r between the first individuals from the two management units are $0.68 + 0.69 - (2 \times 0.55) = 0.27$ and $0.55/(\sqrt{0.68} \times \sqrt{0.69}) = 0.80$, respectively. The result reaffirms that scaling $\mathbf{G}$ to be on the same scale as $\mathbf{A}$, genomic relatedness leads to an increases in the ratio sensitive connectedness statistic, a change consistent with the other statistics. Collectively, these particular examples, suggest that genomic information provided by $\mathbf{G}$ or $\mathbf{G_s}$ changes the estimates of relationship coefficients among animals and refines the estimates of connectedness.

Table S1: Average genetic connectedness statistics across management units in the cattle data. S1 (completely disconnected), S2 (disconnected), S3 (partially connected), and S4 (connected) represent four management unit scenarios. PEVD, CD, and r denote prediction error variance of the difference, coefficient of determination, and prediction error correlation. We combined pedigree-based $\mathbf{A}$ with the standard genome-based $\mathbf{G}$, genome-based $\mathbf{G_{0.5}}$ assuming equal allele frequencies, and scaled genome-based $\mathbf{G_s}$ kernel matrices to evaluate relationships among individuals. Two heritability values 0.8 and 0.2 were simulated.

			Heritability (h^2)	
Scenarios	Methods	Kernels	0.8	0.2
S1	PEVD	$\mathbf{H}$ ($\mathbf{G}$)	0.069	0.095
		$\mathbf{H}$ ($\mathbf{G_s}$)	0.089	0.130
		$\mathbf{H}$ ($\mathbf{G_{0.5}}$)	0.062	0.090
	CD	$\mathbf{H}$ ($\mathbf{G}$)	0.379	0.140
		$\mathbf{H}$ ($\mathbf{G_s}$)	0.473	0.232
		$\mathbf{H}$ ($\mathbf{G_{0.5}}$)	0.504	0.283
	r_{ij}	$\mathbf{H}$ ($\mathbf{G}$)	-0.012	-0.004
		$\mathbf{H}$ ($\mathbf{G_s}$)	0.054	0.036
		$\mathbf{H}$ ($\mathbf{G_{0.5}}$)	0.066	0.060
S2	PEVD	$\mathbf{H}$ ($\mathbf{G}$)	0.014	0.021
		$\mathbf{H}$ ($\mathbf{G_s}$)	0.019	0.028
		$\mathbf{H}$ ($\mathbf{G_{0.5}}$)	0.014	0.020
	CD	$\mathbf{H}$ ($\mathbf{G}$)	0.464	0.216
		$\mathbf{H}$ ($\mathbf{G_s}$)	0.530	0.287
		$\mathbf{H}$ ($\mathbf{G_{0.5}}$)	0.531	0.306
	r_{ij}	$\mathbf{H}$ ($\mathbf{G}$)	-0.015	-0.004
		$\mathbf{H}$ ($\mathbf{G_s}$)	0.040	0.025
		$\mathbf{H}$ ($\mathbf{G_{0.5}}$)	0.050	0.041
S3	PEVD	$\mathbf{H}$ ($\mathbf{G}$)	0.011	0.017
		$\mathbf{H}$ ($\mathbf{G_s}$)	0.013	0.021
		$\mathbf{H}$ ($\mathbf{G_{0.5}}$)	0.010	0.015
	CD	$\mathbf{H}$ ($\mathbf{G}$)	0.510	0.251
		$\mathbf{H}$ ($\mathbf{G_s}$)	0.589	0.344
		$\mathbf{H}$ ($\mathbf{G_{0.5}}$)	0.521	0.265
	r_{ij}	$\mathbf{H}$ ($\mathbf{G}$)	-0.011	-0.003
		$\mathbf{H}$ ($\mathbf{G_s}$)	0.042	0.026
		$\mathbf{H}$ ($\mathbf{G_{0.5}}$)	0.051	0.041
S4	PEVD	$\mathbf{H}$ ($\mathbf{G}$)	0.007	0.007
		$\mathbf{H}$ ($\mathbf{G_s}$)	0.008	0.009
		$\mathbf{H}$ ($\mathbf{G_{0.5}}$)	0.006	0.007
	CD	$\mathbf{H}$ ($\mathbf{G}$)	0.168	0.060
		$\mathbf{H}$ ($\mathbf{G_s}$)	0.255	0.126
		$\mathbf{H}$ ($\mathbf{G_{0.5}}$)	0.256	0.145
	r_{ij}	$\mathbf{H}$ ($\mathbf{G}$)	-0.005	0.001
		$\mathbf{H}$ ($\mathbf{G_s}$)	0.049	0.029
		$\mathbf{H}$ ($\mathbf{G_{0.5}}$)	0.056	0.044

Table S2: Average genetic connectedness measured as coefficient of determination (CD) across management units in the cattle data. S1 (completely disconnected), S2 (disconnected), S3 (partially connected), and S4 (connected) represent four management unit scenarios. We compared pedigree-based **A** with the standard genome-based **G** kernel matrices to evaluate relationships among individuals. Two traits with heritability values of 0.66 (Trait 1) and 0.41 (Trait 2) were analyzed and variance components were estimated from the data rather than assumed known.

		Traits	
Scenarios	Kernels	Trait 1	Trait 2
S1	**A**	0.282	0.200
	**G**	0.345	0.266
S2	**A**	0.336	0.252
	**G**	0.457	0.374
S3	**A**	0.421	0.332
	**G**	0.480	0.395
S4	**A**	0.110	0.081
	**G**	0.213	0.159

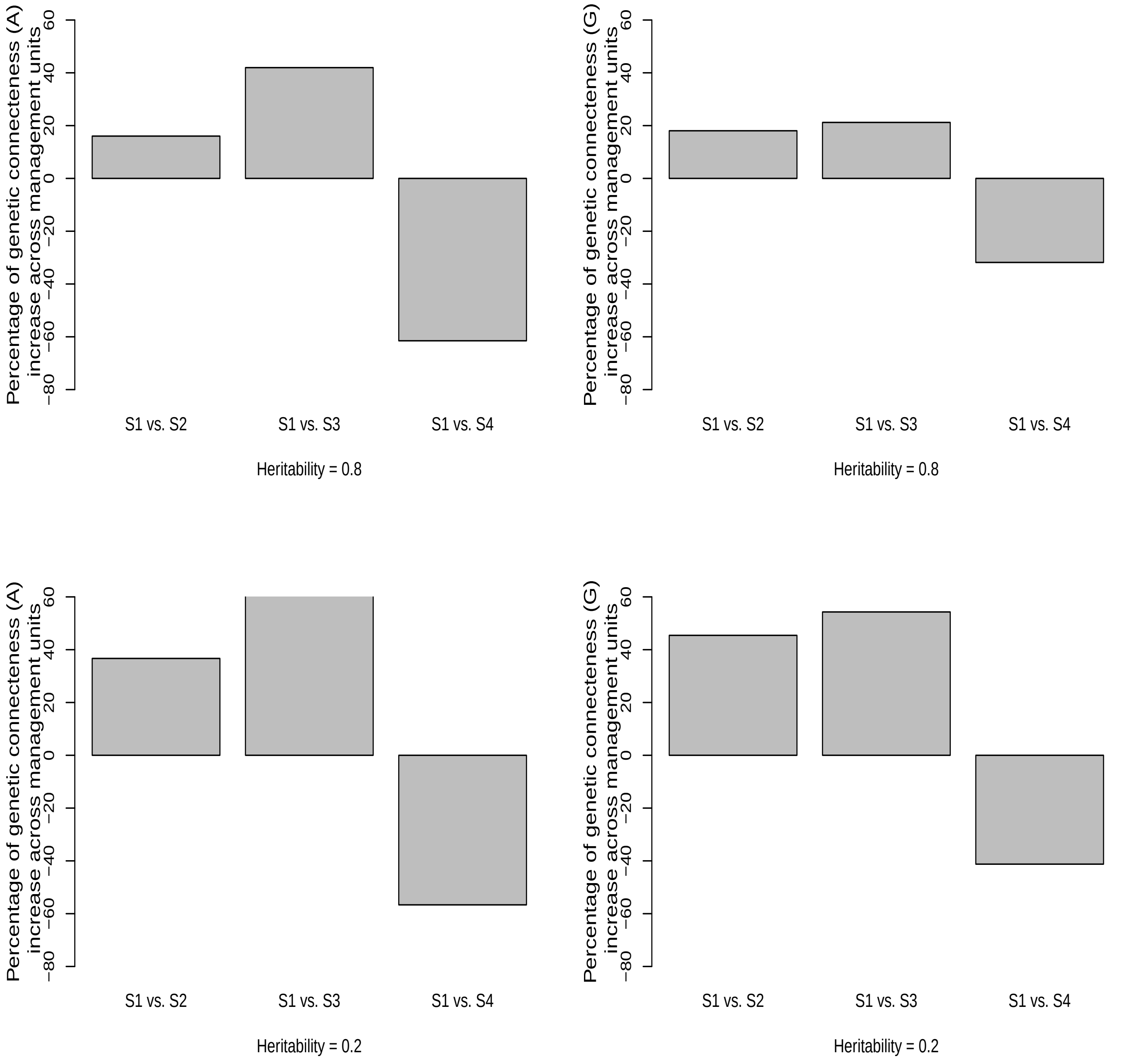

Figure S1: Percentages of relative increase in coefficient of determination of the difference (CD) across management units in comparison to base scenario 1. Two heritability values 0.8 and 0.2 were simulated. S1 (completely disconnected), S2 (disconnected), S3 (partially connected), and S4 (connected) represent four management unit scenarios. Left: **A** matrix. Right: **G** matrix.

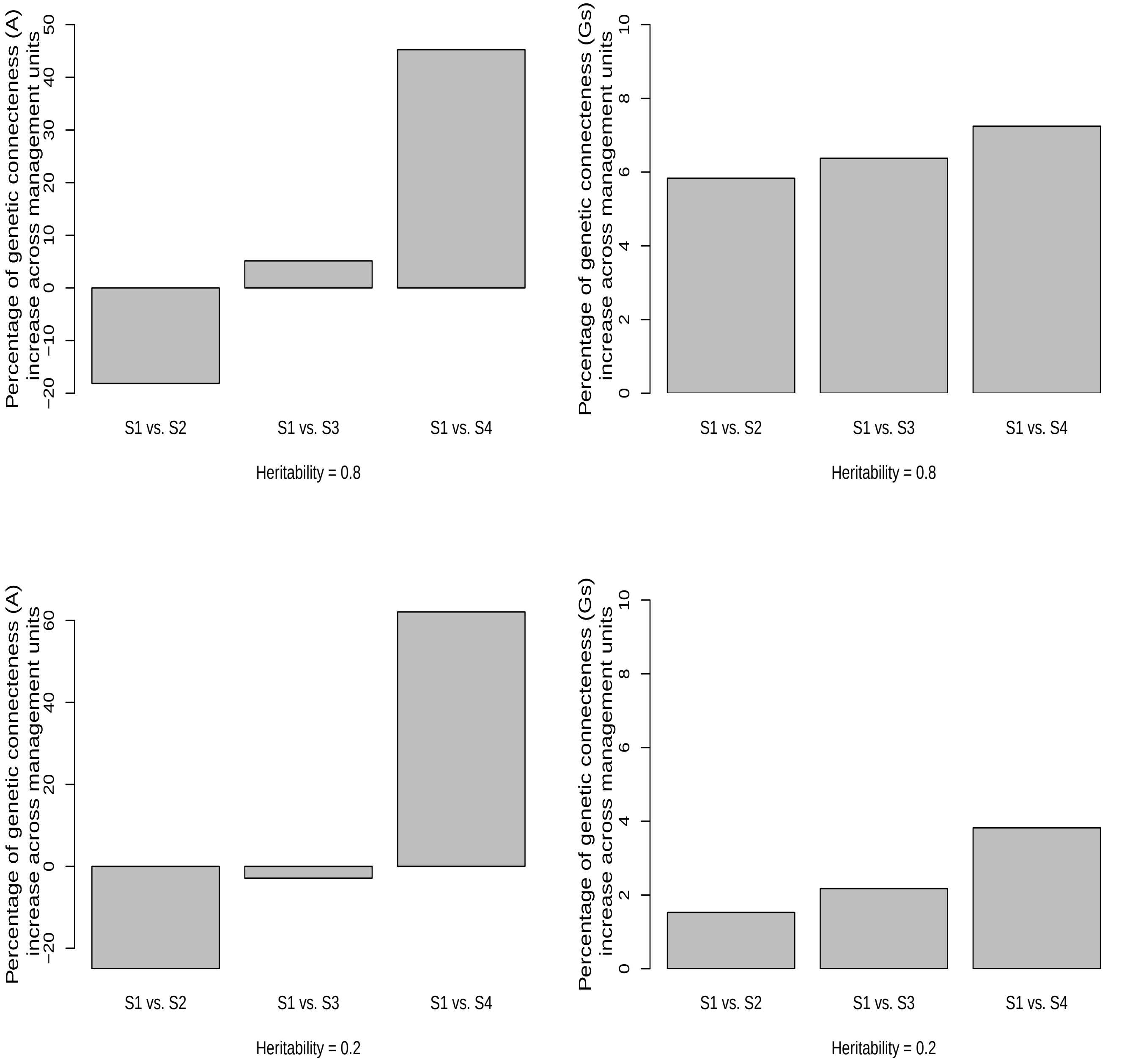

Figure S2: Percentages of relative increase in prediction error correlation (r) across management units in comparison to base scenario 1. Two heritability values 0.8 and 0.2 were simulated. S1 (completely disconnected), S2 (disconnected), S3 (partially connected), and S4 (connected) represent four management unit scenarios. Left: **A** matrix. Right: **Gs** matrix.

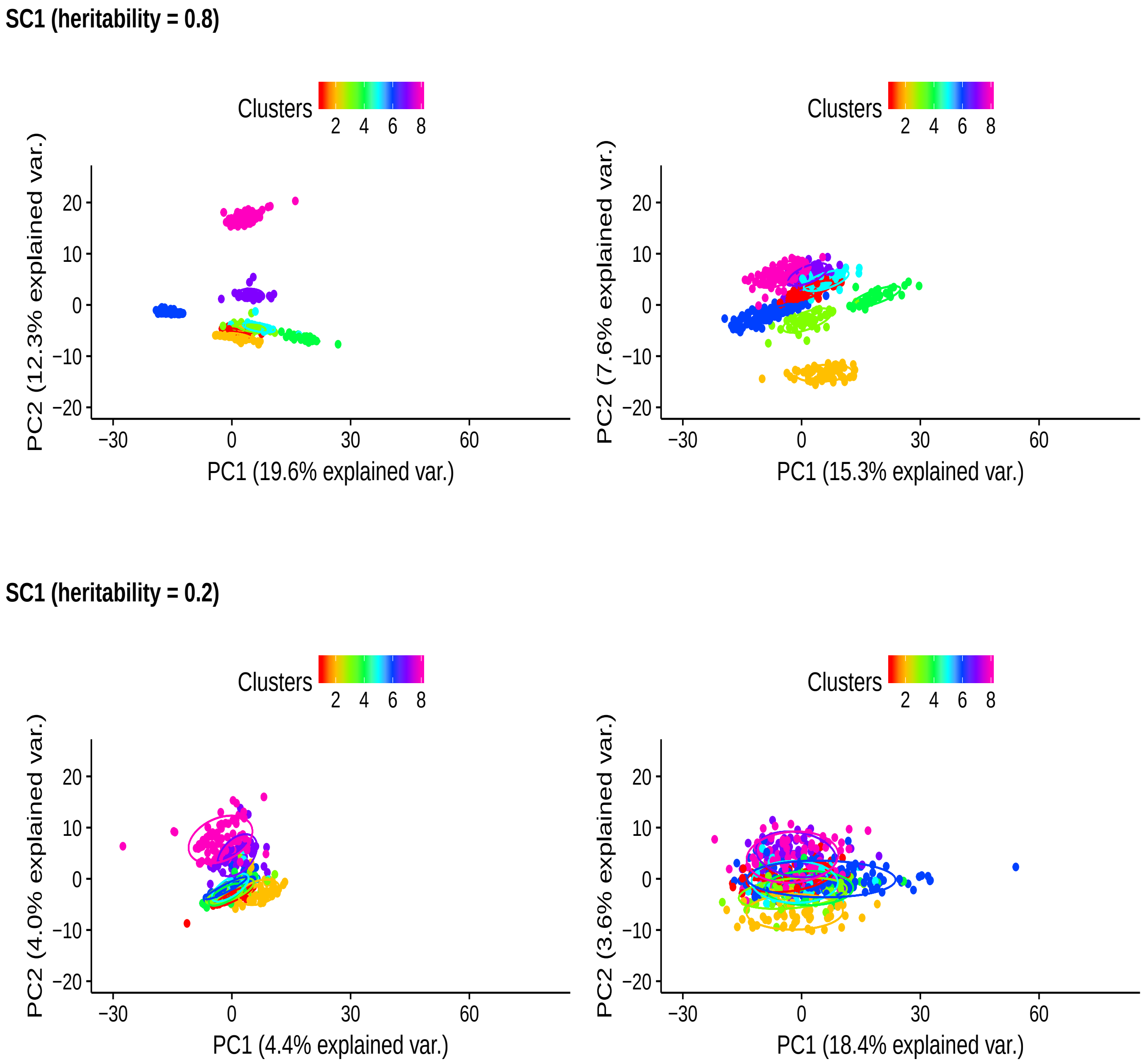

Figure S3: Principle component (PC) plots for Scenario 1 with prediction error variance of the difference (PEVD) statistics. The first and second rows are according to heritability of 0.8 and of 0.2. The first and second columns are derived from pedigree-based and genome-based PEVD, respectively. The PC plots were grouped by clusters and colored in different colors. Individuals within the same cluster were grouped by the circles.

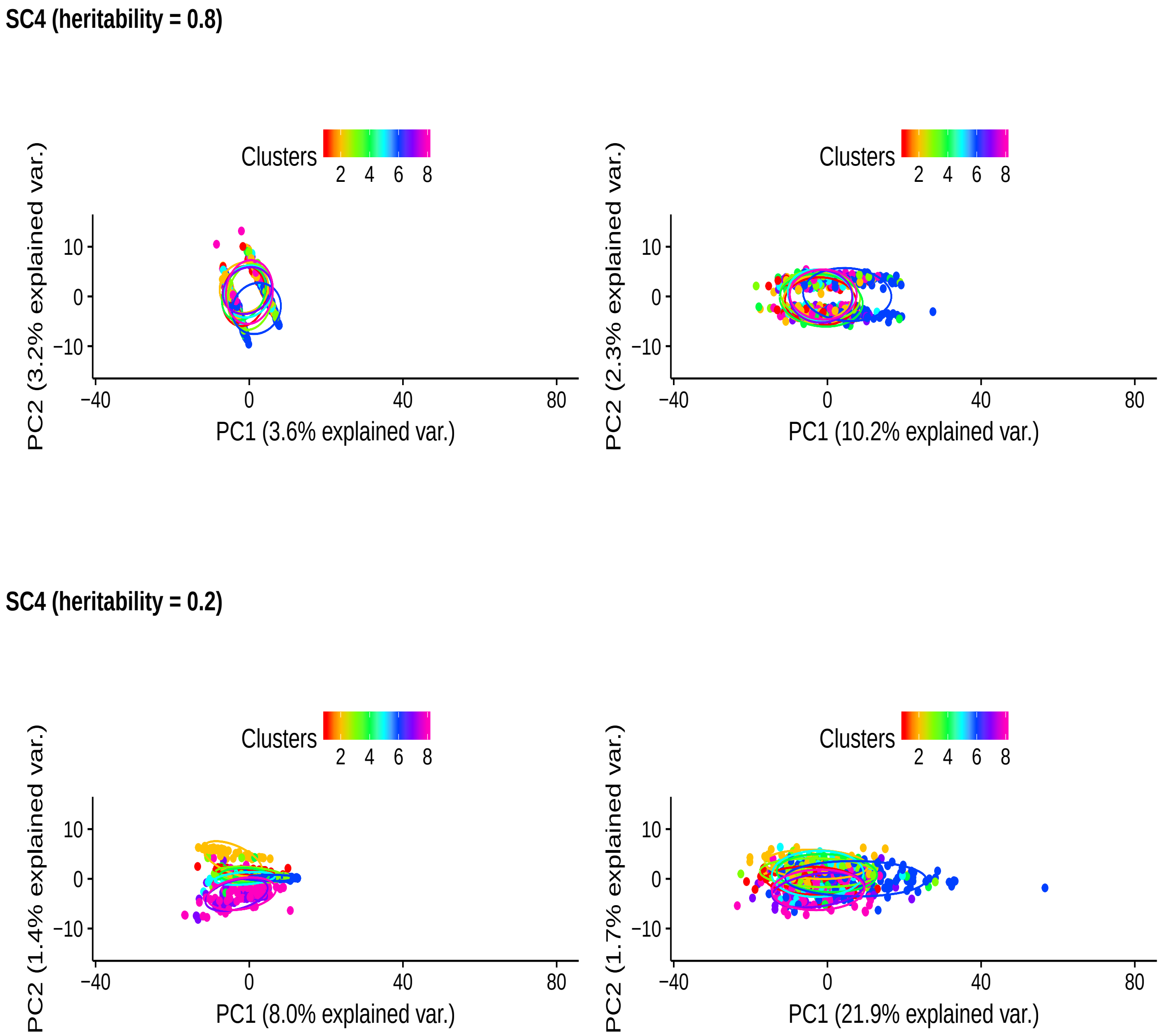

Figure S4: Principle component (PC) plots for Scenario 4 with prediction error variance of the difference (PEVD) statistics. The first and second rows are according to heritability of 0.8 and of 0.2. The first and second columns are derived from pedigree-based and genome-based PEVD, respectively. The PC plots were grouped by clusters and colored in different colors. Individuals within the same cluster were grouped by the circles.

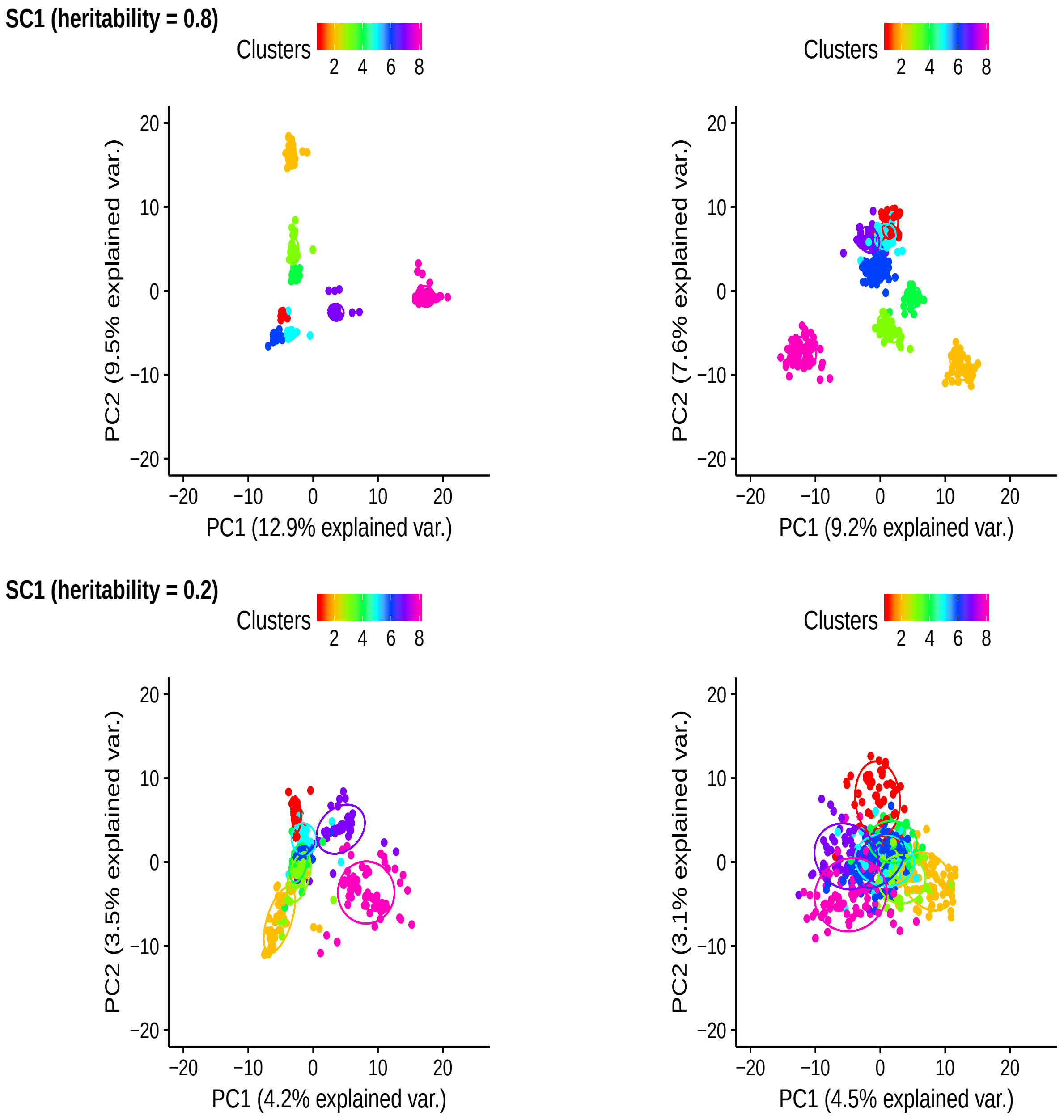

Figure S5: Principle component (PC) plots for Scenario 1 with prediction error correlation (r) statistics. The first and second rows are according to heritability of 0.8 and of 0.2. The first and second columns are derived from pedigree-based and genome-based r, respectively. The PC plots were grouped by clusters and colored in different colors. Individuals within the same cluster were grouped by the circles.

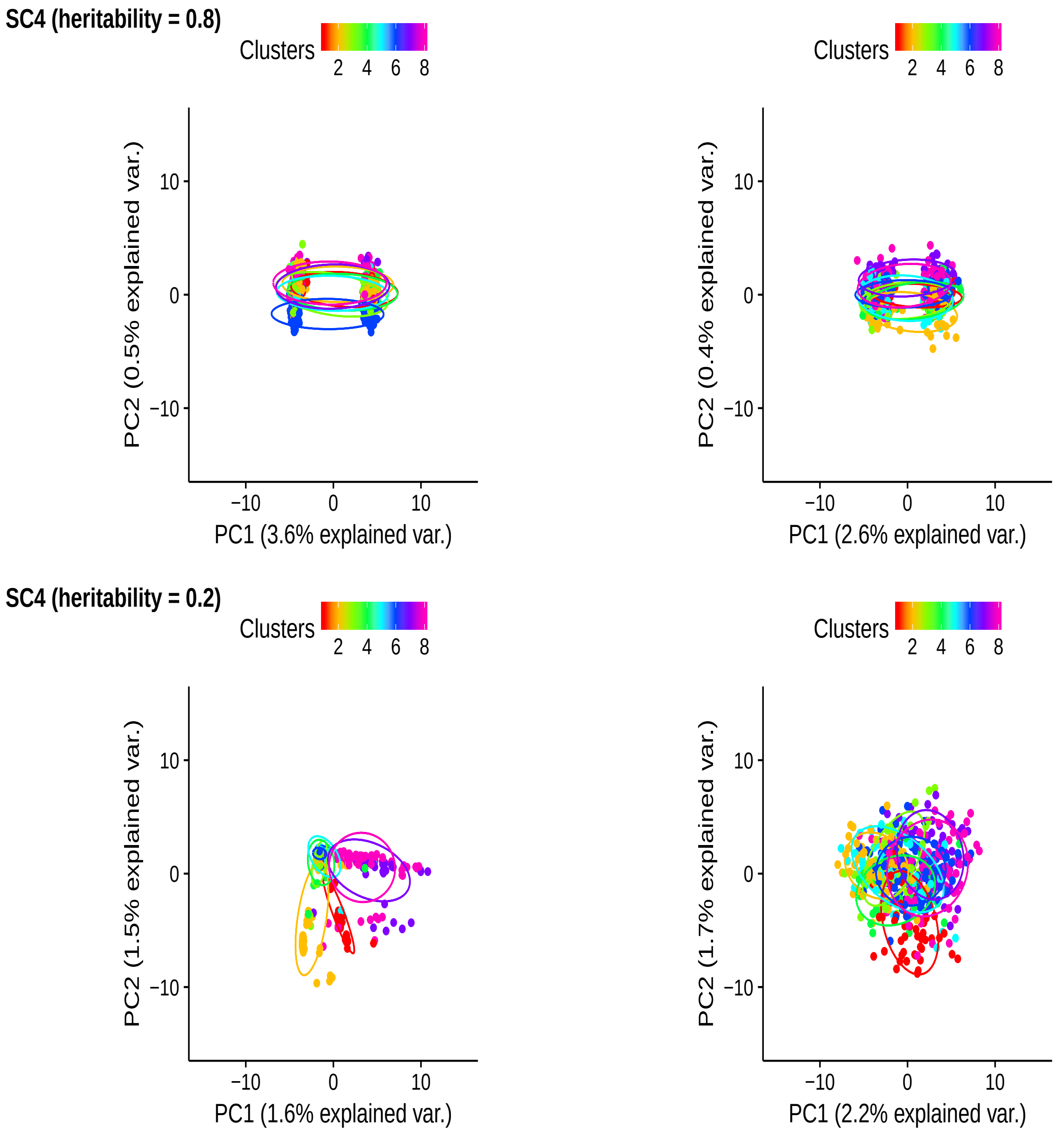

Figure S6: Principle component (PC) plots for Scenario 4 with prediction error correlation (r) statistics. The first and second rows are according to heritability of 0.8 and of 0.2. The first and second columns are derived from pedigree-based and genome-based r, respectively. The PC plots were grouped by clusters and colored in different colors. Individuals within the same cluster were grouped by the circles.
